# Toward Safer and Greener Insulation: Formaldehyde-Free, Flame-Retardant, and Bio-Based Phenolic Foams from Tannin and Modified-Lignin Combination

**DOI:** 10.3390/ma19020334

**Published:** 2026-01-14

**Authors:** Jevgenij Lazko, Jérôme Mariage, Célia Joyet, Abdelheq Layachi, Hamid Satha, Philippe Dubois, Fouad Laoutid

**Affiliations:** 1 Materia Nova Research Center, UMONS Innovation Center, Avenue Nicolas Copernic 3, B-7000 Mons, Belgium; jevgenij.lazko@materianova.be (J.L.); jerome.mariage@materianova.be (J.M.); philippe.dubois@umons.ac.be (P.D.); 2 Laboratoire LSPN, Université 8 Mai 1945 Guelma, BP 401, Guelma 24000, Algeria; abdelhak2416@live.fr (A.L.); sathahamid@yahoo.fr (H.S.); 3 ISTA, Université Frères Mentouri, Constantine 1, Constantine 25000, Algeria

**Keywords:** phenolic foam, lignin, flame retardancy, lignin, tannin, formaldehyde-free

## Abstract

This study reports on the use of degraded lignin in combination with tannins to develop sustainable, formaldehyde-free, and bio-based phenolic foams. Mechanical, thermal, and flame-retardant properties of the different foams were systematically evaluated using compression testing, thermogravimetric analysis (TGA), mass loss cone calorimetry (MLC), and UL-94 flammability tests. Lignin degradation/activation was carried out via a hydrothermal process in the presence of ethanol. Ethanol-induced lignin hydrogenolysis and thermal degradation were deemed a necessary step to obtain foams with satisfactory mechanical, morphological, and thermal insulation properties. Meanwhile, the fire resistance assessed by MLC remains comparable to that of tannin-based foams, with a similarly low peak heat release rate (pHRR).

## 1. Introduction

Environmental concerns related to climate change are driving efforts to reduce greenhouse gas emissions by decreasing the consumption of fossil fuels and promoting the use of renewable materials. Implementing an ambitious policy of thermal insulation for buildings is a key lever to achieve these goals, as it reduces the amount of energy required for regulating the building’s indoor temperature. The adoption of renewable, bio-based insulating materials strengthens this approach by replacing fossil-based materials with sustainable solutions, such as bio-based foams.

Phenolic and polyisocyanurate (PIR) foams are among the most commonly used materials for exterior building insulation. While PIR foams require the addition of flame retardants due to their inherently poor fire resistance, phenolic foams exhibit excellent flame-retardant properties. They are self-extinguishing, do not sustain continuous burning, and produce minimal smoke emissions [1] and no dripping combustion [2]. In addition to these benefits, phenolic foams offer low water absorption, long service life, good resistance to corrosion and acids and even suitability for low-temperature applications (down to −200 °C) [3,4].

Classically, phenolic foams are produced through the reaction between phenol and formaldehyde. However, these primary raw materials present low safety and are derived from non-renewable petroleum-based resources. The development of bio-based alternatives to replace these petroleum-derived products in the preparation of phenolic foams has garnered significant interest. Such efforts aim to improve health and safety during production and use, as well as to reduce the material environmental impact while preserving the desirable properties of the final products.

Several studies have focused on partially replacing phenol with bio-based phenolic derivatives, including lignin [5,6], cardanol and its derivatives [7], and tannins [8]. At the same time, other research efforts have explored less-toxic alternatives to formaldehyde, such as furfural [9] or glyoxal [10].

The use of lignin, a bio-based phenolic source abundantly available as a byproduct of the pulp and paper industries, has garnered significant interest for technical applications. Despite being currently underutilized, lignin presents a valuable opportunity to convert a readily available, cost-effective, and sustainable byproduct into high-value materials. However, lignin presents low reactivity toward formaldehyde, which is largely attributed to its high molecular weight and significant steric hindrance, and thus, cannot be used as it is for the development of phenolic materials. Its degradation and functionalization are required prior to its use in the preparation of phenolic resins [11,12].

Li et al. [3] successfully achieved a high phenol substitution ratio of up to 50% using degraded lignin through a hydrothermal method. The resulting foams demonstrated excellent flame-retardant properties and met the required standards for compressive strength. Phenolation is one of the most common methods used to enhance the reactivity of lignin. This process involves reacting lignin with phenol to incorporate phenol groups into its side chains and aromatic rings [13]. As a result, it increases the number of reactive sites and the content of phenolic hydroxyl groups while reducing its molecular weight. Lignin hydroxymethylation involves introducing methylol groups onto lignin aromatic structure and side chains through a reaction with formaldehyde in an alkaline medium. Both phenolated and hydroxymethylated lignin can then be incorporated into the production of phenol–formaldehyde resins by partially substituting phenol. However, the use of these modified lignins does not eliminate the reliance on petroleum-based and unsafe formaldehyde and phenol.

Using bio-based tannins, alone without phenols and in combination with bio-based alternatives to formaldehyde, represents the most sustainable approach for developing fully bio-based and non-toxic phenolic materials, effectively replacing traditional phenol–formaldehyde-based materials. Due to their high reactivity, condensed tannins are more commonly used in the synthesis of phenolic resins and foams compared to hydrolyzable tannins. Their reaction with formaldehyde is similar to that between formaldehyde and phenol.

N.E. Meikleham and A. Pizzi [14] were the first to develop foams based on condensed tannins. Their method involves a two-step process. First, a mixture of furfuryl alcohol, formaldehyde, water, and mimosa tannin is prepared. Then, diethyl ether is added as a foaming agent, along with toluene-4-sulphonic acid as a catalyst [15]. Furfuryl alcohol plays a key role by generating heat through its exothermic self-condensation reactions and its interaction with tannins. This temperature increase causes the evaporation of diethyl ether, which has a low boiling point (35 °C), leading to the formation of the foam.

Substituting tannins with lignin offers an appealing pathway to valorize an already available byproduct, reduce reliance on tannins specifically extracted from biomass (such as wood and tree bark), and lower the cost of the final product. Such a combination has been investigated for the preparation of phenolic foams [16,17]. Nevertheless, the proportion of lignin in the mixture remained relatively low, with a maximum content of 16.5 wt%.

Based on the aforementioned findings, this work investigates the impact of lignin structure on the production of phenolic foams, achieved by partially substituting condensed tannins at levels exceeding 30 wt%, without relying on either formaldehyde or phenol. Accordingly, we investigated in this study the effect of lignin degradation/activation via a hydrothermal process, used in producing phenolic foams, partially substituting condensed tannins, while taking the approach even further by completely eliminating the use of formaldehyde and phenol. Furfurylic alcohol was used instead of formaldehyde. Lignin was degraded for reducing its molecular size and enhancing its reactivity using a hydrothermal process in the presence of ethanol, following an adapted version of the procedure reported by Kong et al. [18], which was originally designed for depolymerizing lignin to produce aromatic monomers. No additional lignin functionalization steps were applied to minimize energy-intensive and chemically demanding modifications, thereby reducing the environmental impact of the applied treatments. The impact of partially substituting tannins with lignin, at 30 wt% replacement level, on foam morphology, thermal conductivity, mechanical performance, thermal stability, and fire behavior was investigated using a combination of techniques, including scanning electron microscopy (SEM), thermal conductivity measurements, compression testing, thermogravimetric analysis (TGA), mass loss calorimetry (MLC), and UL-94 flammability tests.

## 2. Materials and Methods

### 2.1. Chemicals and Materials

Condensed tannins extracted from seeds of white grapes were supplied from Green’ing company (Bedarieux, France). Sodium Lignin sulfonate from TCI (Tokyo Chemical Industry Co., Ltd., Tokyo, Japan) was purchased from VWR. Ethanol and furfuryl alcohol (>98%) were purchased from Thermo Fisher Scientific (Waltham, MA, USA), while diethyl ether (>99%) and para-toluenesulphonic acid (analytical grade) were purchased from VWR. All additives were used as received.

### 2.2. EtOH-Induced Hydrogenolysis Thermal Degradation of Lignin

Lignin degradation was carried out in a hydrothermal reactor in the presence of ethanol. Typically, a mixture of 15 g of lignin and 150 mL of ethanol was placed in the reactor, which was sealed hermetically and placed in an oven at 200 °C for 1 h or at 215 °C for 4 h. The resulting lignin samples were designated as Lignin 200 °C-1 h and Lignin 215 °C-4 h, respectively.

### 2.3. Foam Preparation Procedure

Phenolic foams were prepared by combining condensed tannin, either alone or blended with lignin, with a cross-linking agent (furfuryl alcohol), a blowing agent (diethyl ether), and an acid catalyst (para-toluene sulphonic acid—PTSA). Typically, 16 g of phenolic bio-based material (tannin alone or mixed with 30 wt% lignin) was combined with 2.4 g of water, 30 g of furfuryl alcohol, and 1.5 g of diethyl ether, and stirred for 20 s. Subsequently, 7.2 g of a 65% aqueous solution of PTSA was added, followed by an additional 20 s of stirring. The resulting mixture was then poured into a mold with the desired shape.

### 2.4. Characterization and Testing

Fourier transform–infrared spectroscopy (FTIR) was employed to compare the structures of lignins obtained after EtOH-induced hydrogenolysis thermal degradation. Spectra were recorded using a Bruker ALPHA II spectrometer (Bruker Optics, Ettlingen, Germany), in the wavenumber range from 4000 cm^−1^ to 400 cm^−1^, with a 2 cm^−1^ resolution and an accumulation of 32 scans.

Potentiometric titration in aqueous solution was employed to assess the carboxyl and phenolic hydroxyl concentrations in the different lignins. Specifically, 0.25 g of lignin was dissolved in 1 mL of deionized water, and the pH was adjusted to above 12 using concentrated NaOH. The solution was continuously stirred until complete dissolution of the sample. Titrations were conducted using 0.1 N HCl until reaching pH 2, utilizing a Metrohm titrator (model 751 GPD Titrino, Metrohm, Herisau, Switzerland).

Scanning Electron Microscopy using XL 20 Phillips field emission gun scanning electron microscope (SEM) (Hitachi high Technologies, Tokyo, Japan) was used for evaluating the effect of the hydrothermal treatment on lignin particle size.

Thermal conductivity measurements were carried out using FP2C conductivimeter (Neotim, Albi, France) by Hot Wire probe method, which is particularly well adapted to characterization of insulating materials. Samples of 10 × 10 × 4 cm^3^ were preconditioned at room temperature, 25 °C, at 50% RH for 48 h. λ measures were performed in triplicate.

Compression testing was carried out on a Lloyd LR10K universal testing machine (Lloyd Instruments LTD, Fareham, UK). A 1 kN load cell with a crosshead speed of 2 mm·min^−1^ and a specimen size of 50 × 50 × 30 mm^3^ was used. The given values are an average of tests carried out on 2 specimens for each foam.

The thermal stability analysis of the different additives and foams was assessed by thermogravimetric analysis (TGA) using TGA II equipment from Mettler Toledo (Greifensee, Switzerland). Approximately 5 mg of the sample was subjected to a temperature ramp from 30 °C to 800 °C at a heating rate of 10 °C·min^−1^ under both air and N_2_.

The flame-retardant behavior of the developed bio-based foams was evaluated using a Mass Loss Cone calorimeter (MLC) and UL-94 tests. MLC tests were performed at a heat flux of 35 kW·m^−2^ using MLC (FTT Ltd., East Grinstead, West Sussex, UK), in accordance with the ISO 17554 standard [19]. The equipment, fitted with a thermopile and chimney for heat release measurements, was used to determine the time to ignition (TTI), peak heat release rate (pHRR), total heat release (THR), and combustion efficiency, defined as the THR at the end of combustion divided by the mass of degraded material. The foams’ ignition resistance and flame-extinguishing properties were evaluated by UL-94 vertical burning tests, using a FIRE apparatus according to the ASTM D3801 standard procedure [20]. Samples measuring 125 × 13 × 3 mm^3^ were subjected to two flame applications (10 s each). The afterflame and afterglow times were measured, and the eventual cotton ignition by flaming drops was recorded.

## 3. Results

### 3.1. Lignin Degradation/Activation

#### 3.1.1. Microscopic Characterization of Treated Lignin Powders

**Figure 1:**
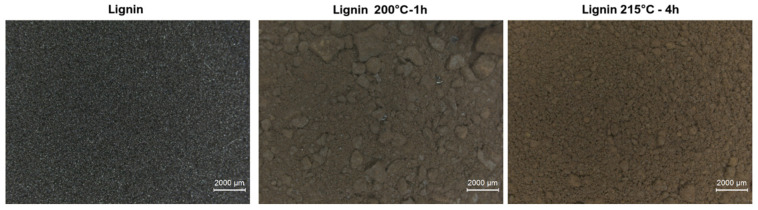
Images of the native lignin and the two grades that underwent the EtOH-induced hydrogenolysis treatment, at different conditions.

Firstly, when lignin undergoes EtOH-induced hydrothermal treatment, a noticeable color change occurs (Figure 1). The untreated lignin is black, whereas the treated lignin, whether exposed to 200 °C for 1 h or 215 °C for 4 h, changes to a brown color. This visible transformation is the first indication of structural modification within the lignin.

**Figure 2:**
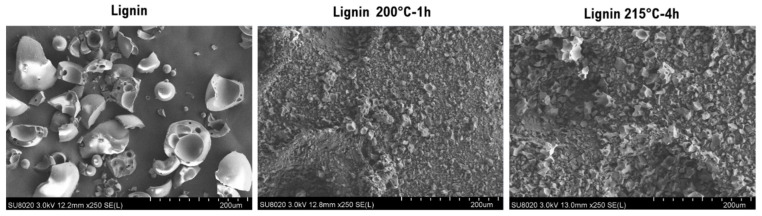
SEM images of lignin powders before and after treatment.

A more detailed SEM analysis of the morphology of the different powders reveals a significant reduction in particle size for the lignins that underwent the treatment (Figure 2). Indeed, the particles in the native lignin exhibit sizes around 100 µm, whereas those in the treated lignins are smaller than 20 µm.

#### 3.1.2. Functionality Evolution

**Figure 3:**
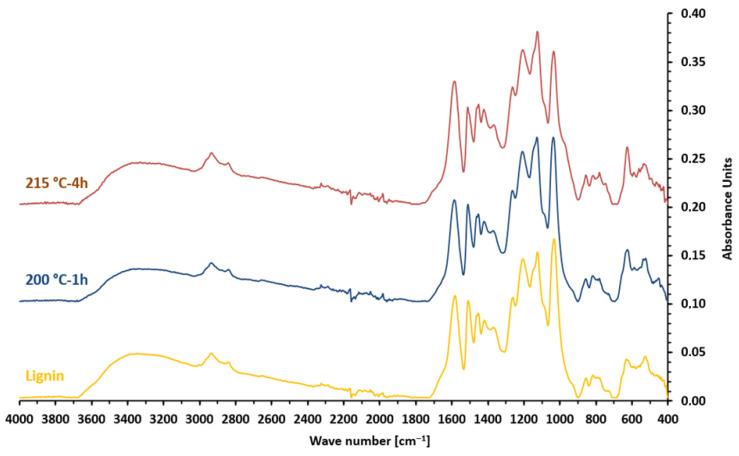
Fourier transform infrared spectrum of native and degraded lignins.

An infrared analysis was conducted to study the effect of lignin hydrothermal treatment on its chemical structure. The Fourier transform–infrared spectra of native lignin and the two degraded ones are presented in Figure 3, and the main peaks along with their assignments are detailed in Table 1. An analysis of the three spectra reveals a strong similarity, indicating that the lignin structure remained unchanged under hydrothermal treatment. The FTIR spectra show no evidence of new peaks, with only the original peaks from the native lignin being detected in the spectra of the two degraded lignins.

To further investigate the effect of EtOH-induced hydrogenolysis thermal treatment on the chemical structure of treated lignins, potentiometric titration was carried out to evaluate the evolution of hydroxyl and carboxyl groups in the different lignin samples.

The results, presented in Table 2, demonstrate that while the COOH content remains stable during treatment, there is a notable decrease in hydroxyl group content. Specifically, concentrations decrease from 4.55 mmol/g in untreated lignin to 2.85 mmol/g and 1.18 mmol/g in lignins treated at 200 °C for 1 h and 215 °C for 4 h, respectively.

The decrease in the number of hydroxyl (OH) groups aligns well with findings reported by Kong et al. [18]. This reduction can be attributed to three primary mechanisms: (1) the preservation of initial methoxy groups that are not degraded during the treatment, (2) the formation of ethoxylated esters potentially resulting from ethanol couplings through an esterification reaction of the degraded aromatics in ethanol, and (3) the dehydroxylation of Cα-OH groups on the aliphatic side chain during ethanol-induced hydrothermal degradation of lignin. However, the effect of the treatment on the molar mass of the treated lignins could not be evaluated by size exclusion chromatography (SEC) due to a change in their solubility after the treatment.

#### 3.1.3. Thermal Degradation

**Figure 4:**
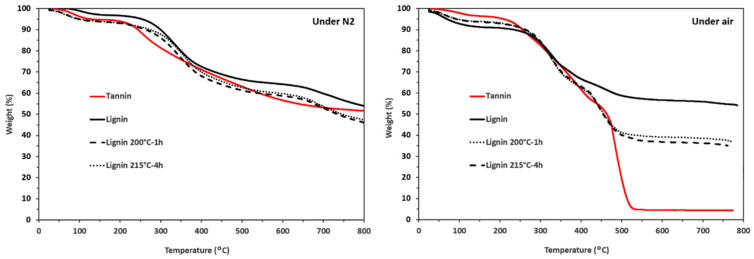
TGA curves of the tannin and lignins, under N_2_ and air, at 10 °C·min^−1^.

The thermal degradation curves of tannin and the various lignins are presented in Figure 4. Under nitrogen, the curves are very similar, highlighting the charring behavior of all four materials. Their degradation occurs gradually, resulting in a char residue of approximately 50% of their initial weight.

However, under air atmosphere, the thermal behavior of the materials begins to diverge above 400 °C, whereas below this temperature, the four degradation curves remain very similar. Beyond 400 °C, tannin undergoes complete decomposition, indicating that the resulting char lacks resistance to thermo-oxidative degradation. In contrast, unmodified lignin yields a higher char residue under the same conditions.

Both thermally treated lignins exhibit comparable degradation behavior, characterized by lower char yields than the original lignin. This reduction in char formation is attributed to the removal of inorganic residues, which, in the case of sodium lignosulfonate, are mainly present as Na_2_O and are eliminated during the thermal treatments applied to obtain the modified lignins (200 °C for 1 h and 215 °C for 4 h).

### 3.2. Foams Properties

The effect of lignin modification on the functional properties of bio-based foams composed of 70% tannin and 30% lignin was systematically investigated. Key characteristics, including morphology, thermal conductivity, compressive mechanical performance, and resistance to thermal degradation and fire, were monitored and analyzed. The results were compared to reference foams formulated either with tannin alone or with 30% unmodified lignin.

#### 3.2.1. Foam Density and Thermal Conductivity

**Figure 5:**
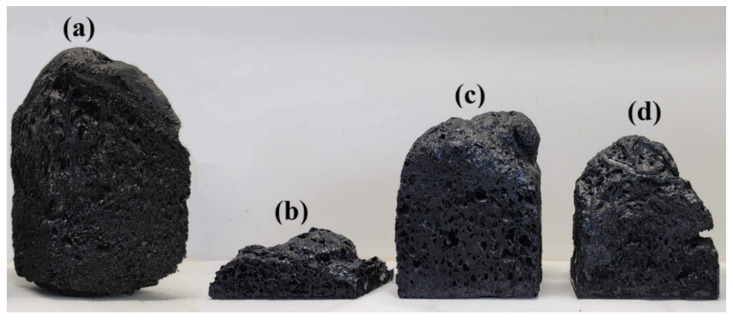
(**a**) 100% Tannin, (**b**) 30% Lignin; (**c**) 30% Lignin 200 °C 1 h, and (**d**) 30% Lignin 215 °C 4 h.

**Figure 6:**
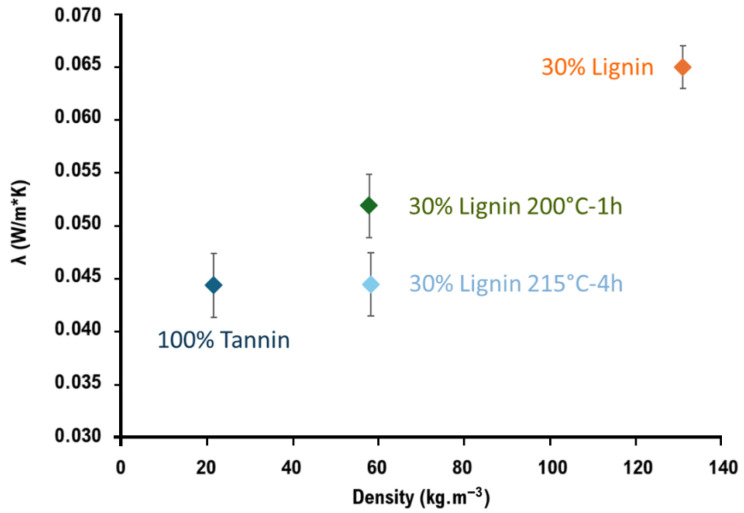
Thermal conductivity as a function of foam density.

Foam density and thermal conductivity are among the key parameters for evaluating the performance of foams used in thermal insulation. The effect of foam composition on both properties is presented in Figure 5 and Figure 6 and Table 3. Several tendencies could be observed concerning the influence of the formulation on the evolution of the density and thermal conductivity of the developed samples.

Foams from tannin alone were the most expanded, with very low densities around 21 kg·m^−3^. On the other hand, the addition of 30 wt% lignin seemed to disturb the foaming process considerably, with the highest densities reaching 130 kg·m^−3^. Lignin hydrothermal treatments had quite positive results on foam development, with samples more than twice as expanded as those with untreated lignin.

Overall, the usual relationship between thermal conductivity and density was observed. The densest samples with untreated lignin were the most conductive with λ being around 0.065 W·m^−1^·K^−1^, while the most expanded tannin-based samples showed the best insulation performances around 0.044 W·m^−1^·K^−1^, which are already close to those of typical commercially available insulating materials (0.039 W·m^−1^·K^−1^). Foams with treated lignins, and especially those modified at 215 °C for 4 h, also exhibited quite interesting thermal conductivity performances. Thus, a λ around 0.045 W·m^−1^·K^−1^ was obtained, even if their density was about 60 kg·m^−3^, probably due to better morphological aspects such as lower porosity size and improved homogeneity and pore distribution.

These results open perspectives for the improvement of the materials’ morphological properties, which are, in fact, the key points for enhancing their insulating performances. The first step could consist of meeting the performances of already commercially available bio-based references, such as insulating materials from natural fibers (λ 0.036–0.040 W·m^−1^·K^−1^), and then, in the second step, targeting λ 0.030 W·m^−1^·K^−1^ values of the most-performant closed-porosity PU foams.

**Figure 7:**
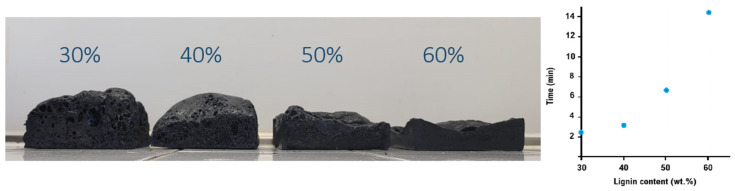
Effect of lignin 200 °C-1 h content on foam expansion (**left**) and foaming time (**right**).

The incorporation rate of 30 wt% of the thermally treated lignin appears to be optimal. Indeed, attempts to formulate new foams with higher lignin contents were unsuccessful, as shown in Figure 7. At a content above 30%, the foam expansion ratio is significantly reduced, while the foaming time increases dramatically, from approximately 2 min with 30% lignin at 200 °C, 1 h. to around 15 min when the lignin content is doubled.

#### 3.2.2. Foam Mechanical Performances

**Figure 8:**
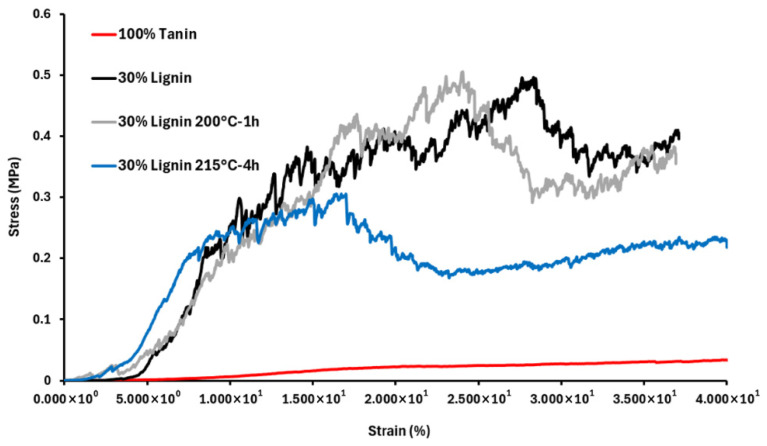
Stress/strain curves of phenolic bio-foams from compression test.

In general, the mechanical performance of foams is related to processing conditions, such as the mixing method, temperature, and reaction time, as well as to foam properties, such as density. It is therefore important to compare the values obtained for our foams with those reported in the literature. Figure 8 presents the compression curves of the investigated foams, and Table 3 summarizes the results for the elastic modulus and compressive strength, in comparison with the values reported for standard phenolic foams by Londono-Zuluaga et al. [12].

It is important to note that the mechanical properties of the 100% tannin-based foam are very poor, primarily due to its low density (21.5 kg·m^−3^), which results from a high expansion rate. In contrast, substituting 30% of the tannin with untreated lignin led to a significant increase in the elastic modulus (19.5 MPa), mainly due to the opposite effect—namely, a drastic reduction in the foam expansion rate, resulting in a much higher density (131 kg·m^−3^). These density values fall below and above, respectively, the typical range reported for standard phenolic foams.

However, the use of thermally treated lignins (200 °C-1 h and 215 °C-4 h) helped to moderate these density variations and yielded foams with densities within the standard range for phenolic foams, along with elastic modulus values comparable to those reported by Lee et al. [21]. Among all formulations, the foam containing 30% of the lignin treated at 200 °C for 1 h exhibited the best mechanical performance, with both high Young’s modulus and compressive strength.

#### 3.2.3. Foam Thermal Degradation

**Figure 9:**
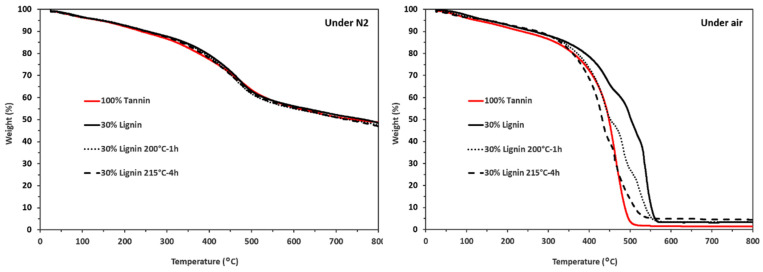
TGA curves of the different phenolic bio-foams, under N_2_ and air, at 10 °C·min^−1^.

The thermal degradation resistance of the four foams was evaluated by thermogravimetric analysis (TGA) under both air and nitrogen atmospheres. The corresponding curves are shown in Figure 9. Under nitrogen, the substitution of 30 wt% of tannin with lignin, regardless of its type, does not alter the thermal behavior, and the final char yields remain comparable.

Under air, some differences are observed in the overall shape of the degradation curves, although no major changes are evident, except when unmodified lignin is used. In this case, after 400 °C, a noticeable deviation occurs, likely due to the presence of mineral residues in the lignin, which may act as reinforcing agents. In contrast, foams incorporating the thermally treated lignins exhibit degradation profiles similar to that of the foam prepared solely with tannin.

#### 3.2.4. Foam Flame-Retardant Properties

**Figure 10:**
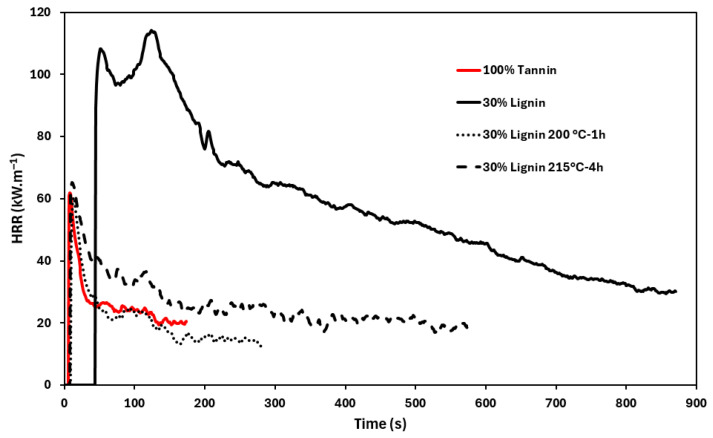
HRR curves of the different bio-based phenolic foams, obtained by mass loss cone calorimeter at 35 kW·m^−2^.

**Figure 11:**
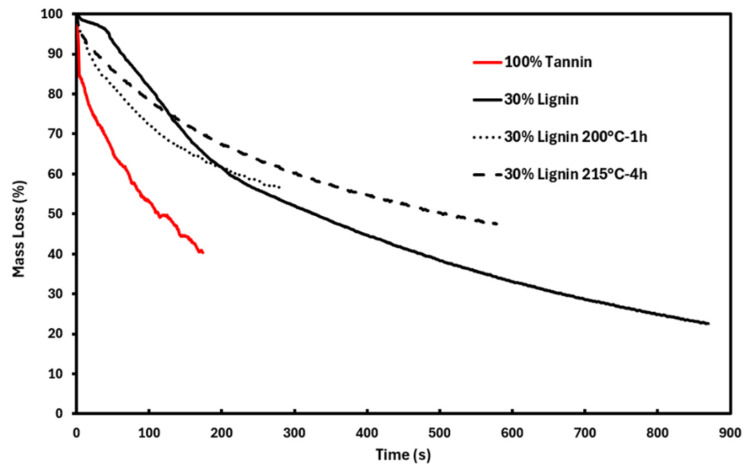
Mass loss rate curves recorded during mass loss calorimetry test.

The fire behavior of the bio-based foams prepared in this study was characterized using both the mass loss calorimetry (MLC) and UL-94 tests (Figure 10 and Figure 11 and Table 4). These complementary methods provide insights into different aspects of the material fire performance. On one hand, MLC assesses the resistance to ignition and heat release of the material during combustion, while the UL-94 test evaluates ignition and flame spread characteristics. These data are crucial for foams intended for use in buildings and homes, where the risk of fire initiation and propagation must be minimized in accordance with current safety standards.

The fire behavior of the foams is significantly influenced by their chemical composition, particularly the nature and proportion of the polyphenolic components. Although the tannin-based foam exhibits limited ignition resistance (TTI = 6 s), this drawback is compensated for by the efficient formation of a protective char layer during combustion. This charring rapidly suppresses the heat release, as evidenced by the low pHRR and the steep decline in the HRR curve following the peak. It is important to note that the fire behavior observed with the 100% tannin-based foam is very similar to what was reported in the literature for phenolic foams [22,23].

Mass loss data provided further insight into the combustion dynamics. The 100% tannin foam undergoes rapid degradation but results in a lower pHRR, suggesting the generation of less-combustible volatile products. In contrast, the foam incorporating 30% unmodified lignin degrades at a slower rate but reaches a higher pHRR, likely due to the higher flammability of lignin-derived species. This delayed degradation aligns with TGA under air, which revealed enhanced thermal stability for the lignin-containing foam.

**Figure 12:**
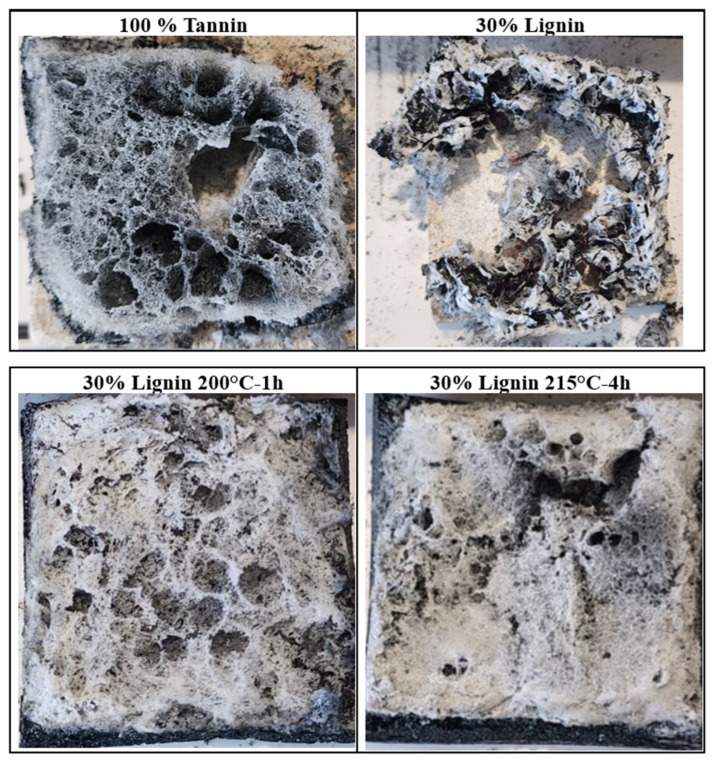
Digital images of the combustion residues obtained after the MLC test of the four foams.

The presence of lignin also negatively affects both the quantity and quality of the combustion residue. Mass loss curves (Figure 11) indicate that only 22% of the initial mass remains at the end of the test for the foam containing 30% lignin, compared to 40% for the 100% tannin-based foam. In addition, the quality of the residual char is significantly altered: while the pure-tannin foam produces a single-piece residue, the foam with 30% lignin results in multiple fragmented debris at the end of combustion (Figure 12). Interestingly, foams incorporating thermally pretreated lignins (200 °C-1 h and 215 °C-4 h), in which lignin is chemically integrated into the foam network, exhibit behavior more akin to the pure-tannin foam. These formulations display a rapid mass loss and reduced ignition resistance, yet maintain a low pHRR, indicating that chemical modification of lignin can tune its fire-related performance. Moreover, both the quality and quantity of the post-combustion residues are improved, with residual masses of 56% and 47% observed for the foams containing lignins treated at 200 °C for 1 h and 215 °C for 4 h, respectively.

Due to the varying densities of the different foams, it is difficult to directly compare the raw THR values, as foam density affects the amount of material exposed during testing. To overcome this, we calculated a new parameter: combustion efficiency, defined as the total heat released (THR) at the end of combustion divided by the mass of degraded material. This parameter reflects how efficiently the degraded material is converted into heat. The combustion efficiency values (Table 4) show that the foam containing 30% undegraded lignin exhibits the highest combustion efficiency (0.7 MJ·m^−2^·g^−1^), compared to lower values ranging between 0.3 and 0.5 MJ·m^−2^·g^−1^ for the three other foams. These results further highlight the advantage of using pre-degraded lignin to develop superior flame-retardant bio-phenolic foams. Moreover, all developed foams exhibited promising fire behavior in the UL-94 test, achieving a V-0 rating with no dripping. None of the four foams ignited or produced flaming drips during the test.

## 4. Conclusions

This study demonstrated the necessity of applying an EtOH-induced hydrothermal treatment, without a catalyst, to thermally activate lignin and facilitate its integration into the phenolic network formed by the combination of condensed tannins and furfuryl alcohol. Without this treatment, lignin does not react with furfuryl alcohol and leads to the formation of a low-expansion foam. In contrast, incorporating lignins activated at 200 °C for 1 h or 215 °C for 4 h significantly improves foam expansion, while enabling better control over the expansion ratio, thermal insulation performances, mechanical properties, and flame-retardant behavior.

## Figures and Tables

**Table 1:** Peaks and assignment of FTIR spectra for native and degraded lignins.

Wave Number (cm^−1^)	Corresponding Group
3335	O-H bonds
2932	CH aliphatic
1582	C=O organic acid salt
1367	OH phenolic
1262	C-O aromatic ether (syringyl)
1205	C-O tertiary alcohol (guaiacyl)
1125	C-O aliphatic ether -O- linkage
1033	C-O primary alcohol or methoxy groups
854	CH aromatic
744	CH aromatic
627	CH deformation

**Table 2:** Results of aqueous titration expressed as mmol of functional groups per g of lignin.

	mmol RCOOH/g	mmol OH/g
Lignin	2.91	4.55
200 °C-1 h	3.06	2.85
215 °C-4 h	3.24	1.18

**Table 3:** Density, thermal conductivity, and mechanical properties of our phenolic foams, in comparison with standard phenolic foam reported in literature [12]. (NR: not reported).

Sample	Density (kg/m^3^)	Thermal Conductivity λ [W·m^−1^·K^−1^]	Elastic Modulus (MPa)	Compression Strength (MPa)
Phenolic foam (standard)	32–120	0.021–0.045	NR	0.10–0.12
100% Tannin	21.5	0.044 ± 0.003	0.43 ± 0.2	0.04 ± 0.01
30% Lignin	131.0	0.065 ± 0.002	19.5 ± 6.6	0.42 ± 0.10
30% Lignin 200 °C-1 h	57.8	0.052 ± 0.003	7.45 ± 1.4	0.45 ± 0.07
30% Lignin 215 °C-4 h	58.2	0.045 ± 0.003	7.3 ± 0.8	0.26 ± 0.06

**Table 4:** Mass loss cone calorimetry and UL-94 test results of the different bio-based phenolic foams.

Sample	TTI (s	pHRR (kW·m^−2^)	THR (MJ.m^−2^)	Combustion Efficiency (MJ·m^−2^·g^−1^)	UL-94
100% Tannin	8 ± 2	47 ± 13	2.5 ± 0.4	0.26	V-0
30% Lignin	43 ± 9	107 ± 7	31 ± 9	0.53	V-0
30% Lignin 200 °C-1 h	9 ± 3	52 ± 5	4.3 ± 0.7	0.22	V-0
30% Lignin 215 °C-4 h	11 ± 5	45 ± 15	4.8 ± 0.3	0.16	V-0

## Data Availability

The original contributions presented in this study are included in the article. Further inquiries can be directed to the corresponding author.

## Abbreviations

The following abbreviations are used in this manuscript:

TGA	Thermogravimetric analysis
MLC	Mass loss cone calorimeter
pHRR	Peak heat release rate
PIR	Polyisocyanurate
SEM	Scanning electron microscopy
PTSA	Para-toluene sulphonic acid
FTIR	Fourier transform–infrared spectroscopy
TTI	Time to ignition
THR	Total heat release
PU	Polyurethane
